# Supplementary figures and images for: Alterations in the Microbiome of Horses Affected with Fecal Water Syndrome
Source: Vet Sci. 2025 Jul 31;12(8):724. doi: 10.3390/vetsci12080724 (PMC12390410; doi:10.3390/vetsci12080724)

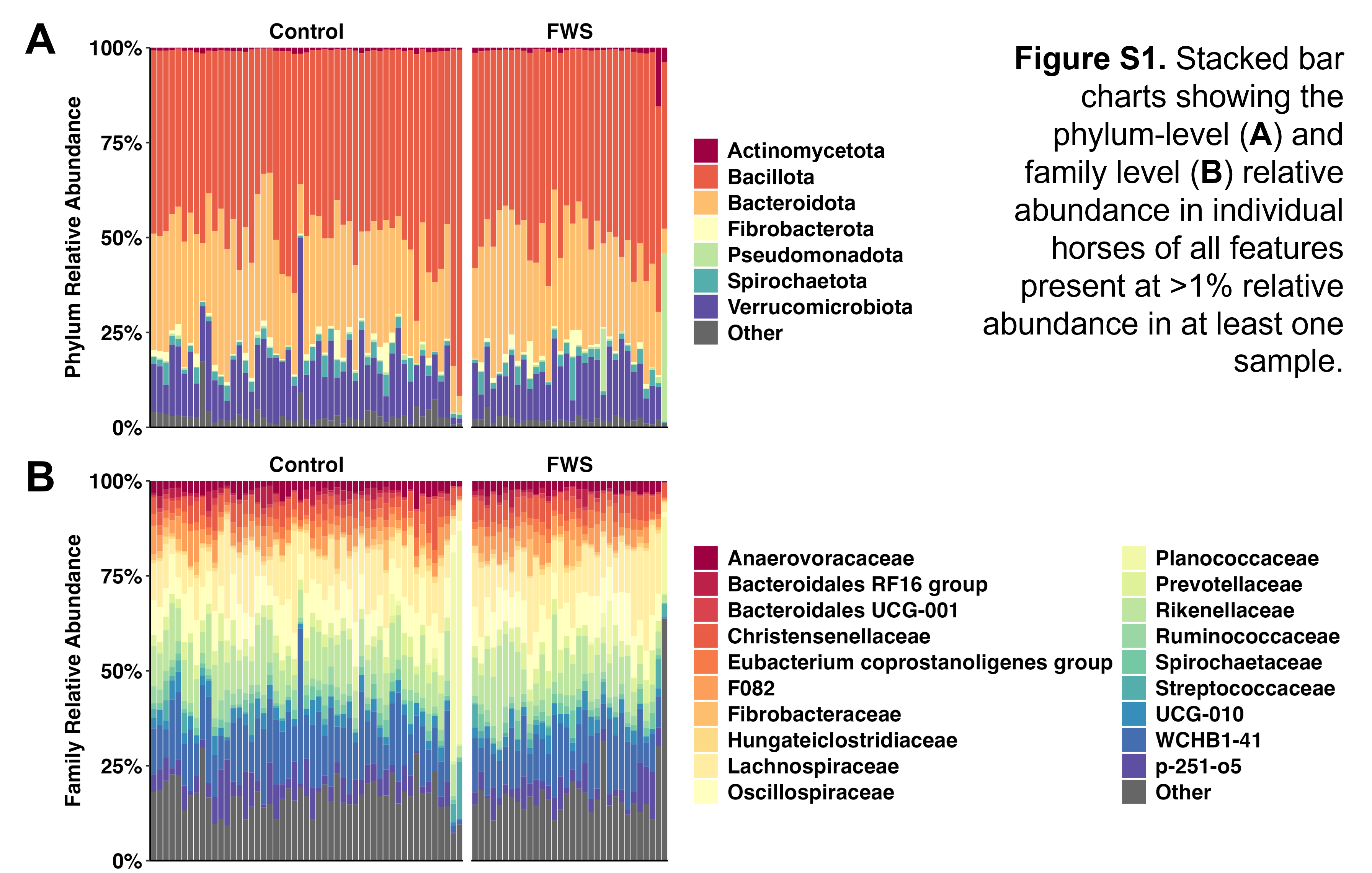

Supplement: Supplementary file 1 [file vetsci-12-00724-s001.zip › Supplemental_material/FigureS1.tif]
